# Short-term blood pressure variability as a potential therapeutic target for kidney disease

**DOI:** 10.1186/s40885-023-00248-3

**Published:** 2023-08-15

**Authors:** Ye Eun Ko, Jong Hyun Jhee

**Affiliations:** 1 Department of Internal Medicine, Yonsei University College of Medicine, Seoul, Republic of Korea; 2 Division of Nephrology, Department of Internal Medicine, Gangnam Severance Hospital, Yonsei University College of Medicine, Seoul, Republic of Korea

**Keywords:** Blood pressure, Ambulatory blood pressure monitoring, Hypertension, Chronic renal insufficiency

## Abstract

Short-term blood pressure variability (BPV) measured with ambulatory blood pressure (BP) monitoring has been demonstrated to be significant in predicting various clinical outcomes. Short-term BPV is distinguished from long-term BPV based on the time interval in which BP fluctuations are measured. Increased short-term BPV has been linked to detrimental effects on the microvascular structure and contributes to subclinical organ damage in the heart, blood vessels, and kidneys, regardless of the average 24-h BP levels. Short-term BPV can be defined by various measures, including calculated metrics (standard deviation, coefficient of variation, average real variability, weighted standard deviation, variability independent of the mean) or dipping patterns. Nevertheless, the additional role of short-term BPV beyond the predictive value of average 24-h BPs or established risk factors for cardiovascular disease and kidney disease remains unclear. In particular, longitudinal studies that evaluate the association between short-term BPV and kidney function impairment are limited and no conclusive data exist regarding which short-term BPV indicators most accurately reflect the prognosis of kidney disease. The issue of how to treat BPV in clinical practice is another concern that is frequently raised. This paper presents a review of the evidence for the prognostic role of short-term BPV in kidney outcomes. Additionally, this review discusses the remaining concerns about short-term BPV that need to be further investigated as an independent risk modifier.

## Background

Blood pressure (BP) has a distinct feature of fluctuating over time, which is referred to as blood pressure variability (BPV). BPV can be classified into two types: short- and long-term BPV. Short-term BPV, which occurs within 24 h and includes minute-to-minute, hourly, and circadian changes, is typically measured by ambulatory BP monitoring (ABPM). In contrast, long-term BPV occurs over longer periods such as days, weeks, months, seasons, or years [1]. The physiological mechanisms proposed to BPV include alterations in ventilation, sympathetic drive [2, 3], the circadian rhythm of cortisol secretion [4, 5], increased blood vessel sensitivity to norepinephrine, diurnal activity pattern for renin and aldosterone (peaking at 8 am, with gradual decrease during the day, nadir at 4 pm) [5–8], and physical activity or sleep patterns [9–11].

Hypertension is well-known risk factor to adverse kidney outcomes, including the development of chronic kidney disease (CKD) in general population with normal kidney function, and the progression of CKD to end-stage kidney disease (ESKD) [12]. Studies including post-hoc analyses of clinical trials and observational studies, have shown that BPV is associated with kidney outcomes beyond average 24-h BP levels [13]. Prior studies have demonstrated an association between short-term BPV and kidney damage [14–17]. Recently, several longitudinal studies have reported that change in short-term BPV increased the risk of adverse kidney outcomes [17–19]. However, several issues remain regarding the proper measurement of short-term BPV and whether it can be treated in practice [20–23].

In this review, the associations between short-term BPV and target organ damage and kidney outcome are discussed, especially incident CKD and CKD progression. This review also addresses various methods used to measure short-term BPV including calculated metrics, dipping pattern, and morning or nocturnal BP. Finally, the pros and cons of using short-term BPV as a prognostic marker for kidney outcome are discussed.

### Mechanism and assessment of short-term BPV

Short-term BPV refers to fluctuations in BP readings over a relatively short period of time, particularly within 24 h [13]. Short-term BPV is a natural physiological response to various environment stimuli or behaviors, including physical activity, postural changes, nonrapid eye movement sleep, and psychological stress [9–11, 24]. The mechanisms of short-term BPV are not fully understood; however, it is thought to result from complex interactions of various physiological and environmental factors. Key mechanisms that contribute to short-term BPV include variability in autonomic nervous system activity such as α-sympathetic vasoconstrictor activity in the morning, which is associated with changes in the waking-sleeping cycle [2, 3], reflex modulation originating from the cardiopulmonary system [25–27], circadian rhythm of hormone excretion such as norepinephrine [5–8], cortisol [4, 5], renin, angiotensin II [28], atrial natriuretic peptide, endothelin-1, bradykinin, nitric oxide, and insulin; changes in vascular tone or blood viscosity [28–31], and several environmental factors.

Short-term BPV can be assessed using noninvasive, intermittent 24-h ABPM with readings taken every 15 to 20 min. Short-term BPV can be evaluated using either calculated metrics or dipping patterns.

### Calculated metrics

Short-term BPV is frequently expressed by the standard deviation (SD) of mean BP values over 24 h [25, 32]. To consider the relationship between the SD and the average BP, the coefficient of variation (CV) is calculated i.e., SD × 100 / mean BP [33]. However, even with these adjustments, these indices are still affected by short-term BP variations and specific stressors such as pain, posture, and emotional stress. Therefore, to avoid such interference, alternative indices such as the average real variability (ARV) weighted for the time interval, which is the mean value of the absolute differences of consecutive measurements, have been introduced to assess rapid BP changes and short-term BPV measurements, as follows [34, 35]:

$$ARV=\frac{1}{\sum t}{\sum }_{a=1}^{n-1}{t}_{a}\times \left|{BP}_{a+1}-{BP}_{a}\right|$$

“a” ranges from 1 to n – 1; “t” is the time difference between BP_a_ and BP_a + 1_; “n” is the number of BP measurements. In addition, weighted SD (wSD) of 24-h BP, which is the average of the daytime and nighttime SDs corrected for the time interval of the daytime and nighttime interval, has been proposed to eliminate the impact of nighttime BP decrease on 24-h SD [36, 37]:

$$wSD=\frac{\left(daytime\,SD \times daytime\,hours\right)+(nighttime\,SD \times nighttime\,hours)}{daytime\,hours+nighttime\,hours}$$

Lastly, variance independent of the mean has been suggested to eliminate the influence of mean BP on BPV using nonlinear regression analysis, such as plotting the relationship between SD and mean BP [38]. When compared with SD and CV, these new methods appeared to correlate better with 24-h BPV [38, 39], which are also better predictors of cardiovascular outcomes [24, 35, 36, 40].

### Dipping patterns and morning BP surge

In the general population, there is a circadian variation in BP, with the highest midmorning BP at 10 am and lowest at 3 am [41]. In the morning BP surges due to an increase in α-sympathetic vasoconstrictor activity [2]. The morning BP surge is typically evaluated through ABPM as the difference between the lowest BP value recorded during nighttime and the highest BP value measured shortly after awakening. In contrast, the nocturnal BP physiologically decreases compared to the diurnal BP since there are less environmental stimuli, emotional stress, and physical activity during the nighttime [9–11]. This normal nocturnal BP decrease is called the “dipping” phenomenon, with night to day BP ratio ranging from 0.80 to 0.90. If the night to day BP ratio is lower than 0.80, the subject is referred to as an “extreme dipper.” However, some patients appear to have reduced nighttime BP dipping (night to day BP ratio > 0.90), which is known as a “nondipping” BP pattern. Some “nondippers” have higher nocturnal than morning BP, thus, they are referred to as “risers,” or “inverted dippers” (night to day BP ratio > 1.0) [42, 43].

### Short-term BPV and kidney outcomes

#### Short-term BPV in experimental study

In experimental studies using spontaneously hypertensive rat (SHR) that underwent sino-aortic denervation (SAD), which induced significant short-term BPV, an association was observed between an increase in short-term BPV and exacerbation of hypertensive renal damages [44, 45]. The large BPV-induced patchy, wedge-shaped, focal sclerotic lesions with interstitial fibrosis and ischemic changes of glomeruli and tubules in the renal cortex in SAD/SHR. Additionally, the afferent arterioles that were adjacent to the cortical sclerotic lesions revealed arteriosclerotic changes characterized by vascular smooth muscle cell proliferation and extracellular matrix deposition, leading to luminal narrowing and occlusion. Notably, the extent of short-term BPV was significantly associated with the area of fibro-ischemic lesions. These findings suggest that the short-term BPV-induced arteriosclerosis and resultant cortical ischemic sclerotic changes in the kidney may contribute to the progression of CKD.

Another study by Freitas et al. [46] examined rat models performed with SAD before inducing CKD via 5/6 nephrectomy. They compared the effects of SAD or CKD alone, and SAD + CKD combined rat models, in terms of alterations in BPV, kidney function, and pathological changes in kidney. Their findings demonstrated that the baroreflex sensitivity index was reduced, while BPV was exacerbated in both SAD and CKD rats, with the most pronounced effects observed in the SAD + CKD rats. With regard to kidney function, the combination of SAD and CKD rates exhibited decreased renal plasma and blood flow, elevated renal vascular resistance, and increased urea levels compared to CKD rats. Moreover, SAD + CKD rats revealed more severe glomerulosclerotic changes, renal hypertrophy, and elevated levels of oxidative stress compared to SAD or CKD alone rats. These results suggest that increased BPV prior to CKD induction by 5/6 nephrectomy aggravates kidney dysfunction and that alteration in short-term BPV may be a contributing factor to the progression of CKD.

#### Short-term BPV and target organ damage

Several studies have demonstrated the relationship between short-term BPV and target organ damage (Table 1) [14–16, 47–62]. Agarwal and Light [55] conducted a cross-sectional study of a sample of 336 general population and showed an association between nondipping patterns and proteinuria. The same investigators extended their studies in patients with CKD and demonstrated that the nondippers are associated with high urine albumin to creatinine ratio (UACR) and low estimated glomerular filtration rate (eGFR) [53]. Similarly, the nondippers have been shown by Pogue et al. [14] to be associated with proteinuria in CKD patients. Fava et al. [54] found positive relationships between the nondipping patterns and low eGFR. In a cross-sectional study with 328 hypertensive patients, Mulè et al. [15] demonstrated that patients with high ARV more commonly had microalbuminuria. In another cross-sectional study involving 169 hypertensive patients, Leoncini et al. [16] showed that patients with high wSD are associated with microalbuminuria or eGFR < 60 mL/min/1.73 m^2^. Corresponding results were observed in prospective studies. Knudsen et al. [47] prospectively studied 112 diabetes mellitus (DM) patients. After an average follow-up of 9.5 years, patients with high diastolic night to day BP ratio showed an increased risk of progression of microalbuminuria or macroalbuminuria. In another prospective study of 75 type 1 DM patients without hypertension, followed for a mean of 5.3 years, Lurbe et al. [50] demonstrated that the nondippers are related to an increased risk of developing microalbuminuria. Likewise, prospective studies involving 957 and 392 DM patients observed that reverse dipping pattern is associated with an increased risk of progression of microalbuminuria or macroalbuminuria [48, 49].

**Table 1:** Summary of the studies with altered short-term BPV and association with target organ damage

Study	Study design	Study subject	No. of subjects	Indices of BPV	Type of association	Outcome
Agarwal and Light [55]	Cross-sectional	General population	336	Nondipping	Positive	Proteinuria
Agarwal and Andersen [53]	Cross-sectional	CKD	232	Nondipping	Positive	Higher UACR, lower eGFR
Fava et al. [54]	Cross-sectional	Non-HTN	249	Nondipping	Positive	Lower eGFR
Cuspidi et al. [52]	Cross-sectional	HTN	355	Nondipping	Indifferent	Microalbuminuria
Cuspidi et al. [51]	Cross-sectional	HTN	375	Nondipping	Indifferent	Microalbuminuria
Pogue et al. [14]	Cross-sectional	CKD	617	Nondipping	Positive	Proteinuria
Song et al. [56]	Cross-sectional	CKD	823	Nondipping, MBPS	Indifferent with nondipping; positive with MBPS	Lower eGFR
Tanner et al. [57]	Cross-sectional	With/without CKD	1,022	SDdn, ARV	Indifferent	Microalbuminuria or eGFR < 60 mL/min/1.73 m^2^
Sarafidis et al. [58]	Cross-sectional	With/without CKD	16,546	SD, wSD, CV, ARV	Positive	Higher UACR, lower eGFR
Ryu et al. [59]	Cross-sectional	CKD	1,173	ARV	Indifferent	Proteinuria and eGFR < 30 mL/min/1.73 m^2^
Wei et al. [60]	Cross-sectional	HTN	256	VIM, ARV	Indifferent	Higher UACR
Mulè et al. [15]	Cross-sectional	HTN	328	ARV	Positive	Microalbuminuria
Madden et al. [61]	Cross-sectional	General population	1,207	wSD, CV, ARV	Indifferent	Microalbuminuria
Farrag et al. [62]	Cross-sectional	HTN	90	SD, CV, ARV	Positive	Microalbuminuria
Leoncini et al. [16]	Cross-sectional	HTN	169	wSD	Positive	Microalbuminuria or eGFR < 60 mL/min/1.73 m^2^
Knudsen et al. [47]	Longitudinal	DM	112	Diastolic night to day BP ratio	Positive	Progression of microalbuminuria, macroalbuminuria
Lurbe et al. [50]	Longitudinal	Non-HTN T1DM	75	Nondipping	Positive	Microalbuminuria
Palmas et al. [48]	Longitudinal	DM	957	Reverse dipping	Positive	Progression of microalbuminuria, macroalbuminuria
Palmas et al. [49]	Longitudinal	DM	392	Reverse dipping	Positive	Progression of microalbuminuria, macroalbuminuria

*BPV* Blood pressure variability, *CKD* Chronic kidney disease, *UACR* Urine albumin to creatinine ratio, *eGFR* Estimated glomerular filtration rate, *HTN* Hypertensive, *MBPS* Morning blood pressure surge, *SDdn* Day-night standard deviation, *ARV* Average real variability, *SD* Standard deviation, *wSD* weighted standard deviation, *CV* Coefficient of variation, *VIM* Variability independent of mean, *DM* Diabetes mellitus, *BP* Blood pressure, *T1DM* Type 1 diabetes mellitus

#### Short-term BPV and incident CKD

To date, few prospective longitudinal studies have been performed to evaluate the association between short-term BPV and incident CKD risk (Table 2) [17–19, 63, 64]. An et al. [63] evaluated 102 individuals in the general population and showed that nondipping patterns were associated with new occurrence of eGFR < 60 mL/min/1.73 m^2^ or UACR > 30 mg/gCr. Similarly, McMullan et al. [18] prospectively studied the general population. After a median follow-up of 8.1 years, subjects with a nondipping pattern showed an increased risk of new occurrence of eGFR < 60 mL/min/1.73 m^2^ or annual decline in eGFR. In addition, Cho et al. [19] have shown that nondipping and reverse dipping patterns were associated with an increased risk of new occurrence of eGFR < 60 mL/min/1.73 m^2^ or UACR > 30 mg/gCr. In a prospective study of 1,173 hypertensive patients, Jhee et al. [17] demonstrated that high ARV is related to new occurrence of eGFR < 60 mL/min/1.73 m^2^, 30% decline in eGFR from baseline, or a new occurrence of urine protein to creatinine ratio > 300 mg/gCr. In another prospective study with 622 hypertensive patients, Turak et al. [64] showed that morning blood pressure surge increased the risk of new occurrence of eGFR < 60 mL/min/1.73 m^2^. Despite the paucity of data on incident CKD and its association with short-term BPV, recent research findings and the physiological mechanism of BPV indicate that short-term BPV is an important risk factor for incident CKD. Thus, additional studies are warranted to consolidate this evidence.

**Table 2:** Summary of the longitudinal studies with altered short-term BPV and association with incident chronic kidney disease

Study	Study subject	No. of subjects	Indices of BPV	Type of association	Outcome
An et al. [63]	General population	102	Nondipping	Positive	New occurrence of eGFR < 60 mL/min/1.73 m^2^ or UACR > 30 mg/gCr
Cho et al. [19]	HTN	995	Nondipping or reverse dipping	Positive	New occurrence of eGFR < 60 mL/min/1.73 m^2^ or UACR > 30 mg/gCr
Jhee et al. [17]	HTN	1,173	ARV	Positive	New occurrence of eGFR < 60 mL/min/1.73 m^2^, 30% decline in eGFR from baseline, or new occurrence of UPCR > 300 mg/gCr
McMullan et al. [18]	General population	603	Nondipping	Positive	New occurrence of eGFR < 60 mL/min/1.73 m^2^ or annual decline in eGFR
Turak et al. [64]	HTN	622	MBPS	Positive	New occurrence of eGFR < 60 mL/min/1.73 m^2^

*BPV* Blood pressure variability, *eGFR* estimated glomerular filtration rate, *UACR* Urine albumin to creatinine ratio, *HTN* Hypertensive, *ARV* Average real variability, *UPCR* Urine protein to creatinine ratio, *MBPS* Morning blood pressure surge

#### Short-term BPV and progression of CKD

Studies on short-term BPV and its association with CKD progression in CKD patients are summarized in Table 3 [20, 65–75]. Patients with CKD are more susceptible to alterations in short-term BPV. In patients with CKD, changes in BPV are caused by increased sodium [76] and fluid retention [77], baroreceptor dysfunction [78–86], altered sympathetic nervous system activity [78–80, 82, 83, 85, 87], renin-angiotensin system activation [87, 88], endothelial dysfunction [89–95], inflammation [96–98], and increased oxidative stress [99, 100]. In a prospective study of 906 hypertensive CKD patients, patients with a nondipping pattern showed an increased risk of ESKD requiring dialysis and an eGFR decline > 50% [71]. Similarly, in another prospective study of 322 patients with CKD, Davidson et al. [72] demonstrated the association between nondipping and faster decline in eGFR after 3.2 years of follow-up. Likewise, in a prospective study of 470 CKD patients, Jhee et al. [73] demonstrated that higher ARV is associated with an increased risk of rapid eGFR decline after 4.3 years of follow-up. In another prospective study of 1,421 patients with CKD, Wang et al. [74] demonstrated that a higher wSD is associated with an increased risk of ESKD.

**Table 3:** Summary of the longitudinal studies with altered short-term BPV and association with CKD progression

Study	Study subject	No. of subjects	Indices of BPV	Type of association	Outcome
Agarwal et al. [66]	With/without CKD	217	Nondipping	Indifferent	ESKD
Agarwal et al. [65]	CKD	322	Nondipping	Indifferent	ESKD
Borrelli et al. [71]	CKD	906	Nondipping	Positive	Initiation of dialysis or eGFR decline ≥ 50%
Davidson et al. [72]	CKD	322	Nondipping	Positive	Rapid decline in eGFR
Ida et al. [68]	CKD	1,107	Nondipping	Indifferent	Initiation of RRT or eGFR decline ≥ 40%
Liu et al. [75]	CKD	304	MBPS	Positive	ESKD or eGFR decline ≥ 50%
Borrelli et al.[20]	CKD	465	wSD and CV	Indifferent	ESKD or eGFR decline ≥ 50%
Jhee et al. [73]	CKD	470	ARV	Positive	Rapid kidney function decline^a)^
Rahman et al. [70]	CKD	1,502	Nondipping or reverse dipping	Positive	ESKD or eGFR decline ≥ 50%
Redon et al. [67]	CKD	79	Nondipping	Indifferent	Rapid decline in eGFR^a)^
Wang et al. [69]	CKD	588	Reverse dipping	Positive	ESKD or doubling of serum creatinine
Wang et al. [74]	CKD	1,421	wSD	Positive	ESKD

*BPV* Blood pressure variability, *CKD* Chronic kidney disease, *ESKD* End-stage kidney disease, *eGFR* estimated glomerular filtration rate, *RRT* Renal replacement therapy, *MBPS* Morning blood pressure surge, *ARV* Average real variability, *wSD* weighted standard deviation, *CV* Coefficient of variation

^a)^eGFR decline > 3 mL/min/1.73 m^2^ per year

### Interaction and clinical relevance between short-term BPVs by different estimates

While both dipping pattern and calculated short-term BPV metrics are relevant in assessing clinical outcome risks, they represent distinct concepts [101]. Dipping pattern refers to the natural physiological phenomenon in which BP decreases during sleep compared to daytime levels. On the other hand, short-term BPV metrics, which are calculated by various methods, refer to the variation in BP readings over a 24-h period. Short-term BPV metrics can be caused by various factors such as changes in physical activity, stress, or medications. Although both have clinical implication assessing target organ damage or poor renal outcomes, complicating results are reported. Recent study by Jhee et al. [17] used three different metrics to define short-term BPV (ARV, SD, and CV). They found that higher short-term BPV by ARV was only associated with higher risk of incident CKD among hypertensive patients. They also found that dipping status had no significant association with incident CKD. However, other studies using dipping pattern observed significant association with incident CKD among general population with or without hypertension [18, 19, 63]. In CKD patients, the relationship between the dipping pattern or short-term BPV metrics and renal outcome is much uncertain, with conflicting results reported [65–72]. The findings of a systemic review and meta-analysis revealed a significant association between ARV and an increased risk of target organ damage [102]. ARV demonstrates distinct advantages compared to other short-term BPV metrics and dipping pattern, including its ease of calculation, ability to reflect the sequential order of BP measurements, and less sensitivity to normal circadian rhythms. These advantages have prompted some researchers to advocate for ARV to be considered as the standard short-term BPV metric. However, it is unclear which of the indices has the most clinical relevance for prediction renal outcome.

In the study by Jhee et al. [17], a strong correlation was observed between various short-term BPV metrics, specifically ARV and SD. However, it remains unclear how these metrics interact with the changes in BP during daytime and nighttime. Notably, their findings indicated that the correlation between ARV and SD did not differ significantly across dipping patterns. However, interesting finding of this study was that ARV showed independent predictive power even after adjusting for dipping pattern in a risk prediction model for renal outcome. Therefore, whether ARV or other short-term BPV metrics such as SD or CV and dipping pattern better represents renal outcome risk remains unclear.

The wSD is determined based on the individual daytime and nighttime SD values, each of which is weighted by hours in the respective period. The wSD is less affected by dipping pattern, which has led to its evaluation in several studies. Leoncini et al. [16] found that an increase in wSD is associated with microalbuminuria or lower eGFR, while Sarafidis et al. [58] observed a correlation between higher wSD and he with advancing stages of CKD in a larger study population (*n* = 16,546). Wang et al. [74] demonstrated that wSD is significantly associated with an increased risk of ESKD among CKD patients. Despite these findings, wSD has limitations, such as the lack of a standardized method for determining nighttime periods. Furthermore, a study by Borrelli et al. [20] reported negative association between wSD and risk of ESKD. Therefore, additional research is necessary to validate the clinical relevance of wSD as a prognostic index short-term BPV.

### Short-term BPV, kidney function, and clinical applications

As described above, short-term BPV has several potential benefits in terms of kidney health and clinical outcomes. Chronic high BP is a major risk factor for the development of kidney disease [12, 103–105]. Reducing short-term BPV may contribute to reducing strain on the kidney and slowing the progression of kidney disease [106]. In addition, stabilizing short-term BPV may improve the blood flow of the kidney vasculatures, increase oxygen and nutrient delivery, and consequently improve kidney function. Furthermore, given that hypertension is one of the most potent risk factors for kidney disease [12], understanding short-term BPV may enable healthcare providers to better manage an individual’s BP levels, and reduce their risk of unfavorable outcomes.

However, there are several limitations of short-term BPV that are yet to be met in practice. First, there are no gold standard methods for short-term BPV measurement. BP readings assessed at different times or devices may result in conflict values, making it difficult to reliably assess short-term BPV [23]. Furthermore, uncontrolled factors affecting BP reading such as changes in posture, physical activity, and diet [24] make it challenging to assess BPV solely from the underlying illness. Second, although positive associations were shown in previous studies between short-term BPV metrics and target organ damage or renal outcome using categorical and continuous variable, most of studies did not report cutoff value for risk stratification. According to the European Society of Hypertension position paper on BPV, daytime systolic SD BPV > 15 mmHg, nocturnal systolic SD > 12.2 mmHg, diastolic SD > 7.9 mmHg, and systolic wSD > 12.8 mmHg were proposed as markers of increased risk for cardiovascular events and death [107]. Further research is needed to determine cutoff value of short-term BPV metrics regarding renal outcome. Third, as short-term BPV only captures BP fluctuation within 24 h, it cannot reflect the influence of long-term BPV [108]. There is insufficient evidence to determine whether short- or long-term BPVs have a greater influence on kidney health [13], and additional research is required. Finally, although recent studies have shown an association between short-term BPV and kidney outcome risks, the underlying mechanisms have not been fully elucidated [13]. Thus, challenges remain in accurately interpreting the study results.

## Conclusions

**Fig. 1:**
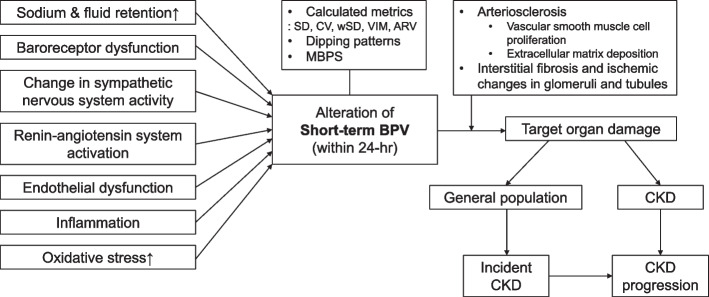
Mechanisms and Impact of Short-term BPV in Kidney Disease

Recent research provides support for the value of short-term BPV in predicting target organ damage and kidney outcomes, such as incident CKD and progression of CKD (Fig. 1). Despite these promising findings, several challenges remain to be addressed, including a limited understanding of the mechanisms through which short-term BPV impacts kidney disease and limitations of measuring methods of short-term BPV. In order to enhance the utility of short-term BPV as a predictive tool in practice, additional evidence is needed regarding its clinical implications of kidney disease.

## Data Availability

Not applicable.

## Abbreviations

ABPM: Ambulatory blood pressure monitoring
ARV: Average real variability
BP: Blood pressure
BPV: Blood pressure variability
CKD: Chronic kidney disease
CV: Coefficient of variation
DM: Diabetes mellitus
eGFR: Estimated glomerular filtration rate
ESKD: End-stage kidney disease
HTN: Hypertensive
MBPS: Morning blood pressure surge
RRT: Renal replacement therapy
SAD: Sino-aortic denervation
SD: Standard deviation
SDdn: Day-night standard deviation
SHR: Spontaneously hypertensive rat
T1DM: Type 1 diabetes mellitus
UACR: Urine albumin to creatinine ratio
UPCR: Urine protein to creatinine ratio
VIM: Variability independent of mean
wSD: Weighted standard deviation
